# Interpretation and applicability of microRNA data to the context of Alzheimer's and age-related diseases

**DOI:** 10.18632/aging.100131

**Published:** 2010-04-02

**Authors:** Patrick Provost

**Affiliations:** CHUL Research Center/CHUQ and Faculty of Medicine, Université Laval, Quebec, QC, G1K 7P4, Canada

**Keywords:** Aging, brain; Alzheimer's disease; microRNA; gene regulation

## Abstract

Generated by the ribonuclease III Dicer, microRNAs (miRNAs) are predicted to regulate up to 90% of the genes in humans, suggesting that they may control every cellular processes in all cells and tissues of the human body! Likely to play a central role in health and disease, a dysfunctional miRNA-based regulation of gene expression may represent the main etiologic factor underlying age-related diseases affecting major organs, such as the brain. Here, we discuss some of the limitations associated to the interpretation and applicability of miRNA data, based on our recent study on the etiology of Alzheimer's disease (AD). Using transiently transfected murine neuronal N2a cells in culture, in parallel to a mouse model of AD, we were able to demonstrate a role for two miRNAs (miR-298 and miR-328) in the regulation of ß-amyloid (Aß) precursor protein (APP)-converting enzyme (BACE) messenger RNA (mRNA) translation, thereby providing key insights into the molecular basis underlying BACE deregulation in AD. However, whether miRNA data can be extrapolated and transposed to the human context of age-related diseases, such as AD, not only requires caution, but also warrants several considerations.

## Introduction

The microRNA (miRNA)-guided RNA silencing pathway is a gene regulatory process present in almost all eukaryotic cells and based on small non-coding RNAs known as miRNAs [1]. Generated by the ribonuclease III Dicer, miRNAs are key regulators of gene expression that act mainly through recognition of specific binding sites generally located in the 3' untranslated region of specific messenger RNAs (mRNAs). Predicted to regulate up to 90% of the genes in humans [2], miRNAs may control every cellular processes in all cells and tissues of the human body! Required for the fine tuning and tight regulation of cellular protein expression, a normal miRNA function is critical for the maintenance of health and prevention of disease [3]. Deregulation of protein expression induced by a dysfunctional miRNA-based regulatory system, which may be either global or miRNA-specific in nature, may thus represent the main etiologic factor underlying age-related diseases, such as Alzheimer's disease (AD) that affects the brain (Provost, manuscript submitted).

### MicroRNAs and Alzheimer's disease

AD is a slowly progressing, age-related neurodegenerative disease that currently affects ~2% of the population in industrialized countries and whose incidence is predicted to increase dramatically over the next 40 years (http://www.alz.org) [4]. Affecting cholinergic neurons, AD is characterized by the accumulation of plaques formed of short ß-amyloid (Aß) peptides in the hippocampal region of the brain [5]. Aß peptides are produced upon proteolytic cleavage of APP by ß-secretase, also known as ß-site APP-cleaving enzyme 1 (BACE1), which contributes to the formation of these plaques [6] (Provost, manuscript submitted).

Post-mortem analyses have revealed upregulation of BACE1 expression at the protein, but not at the mRNA, level in brains from patients suffering from AD, as compared to brains from unaffected patients [7], consistent with an impaired control of BACE1 mRNA translation. In a recent study from our laboratory, we reported similar observations in an animal model of AD (APPSwe/PS1 mice) and demonstrated a role for two miRNAs, i.e. miR-298 and miR-328, in the regulation of BACE1 expression, using mainly transiently transfected murine neuronal N2a cells in culture [8]. In vivo, we observed decreased expression levels of miR-298 and miR-328 in the hippocampus of aging APPSwe/PS1 mice [8], which supports further the possibility that the loss of miRNA regulation of BACE1 mRNA translation may lead to higher BACE1 protein expression, an enhanced Aß formation and the development of AD.

## Experimental considerations

Whether these findings can be extrapolated and transposed to human requires prudence and cautiousness, especially in the context of multifactorial, age-related diseases like AD, which may result from an intricate interplay of genetic and environmental factors. Several additional issues warrant further considerations and need to be taken into account, or addressed, in order to ascertain our interpretation of miRNA data and, most importantly, the transposability of our findings to the aging human beings, such as (i) the nature and source of the biological material under investigation, (ii) the use and relevance of cellular models, (iii) the use of primary versus cultured cells, and (iv) the other functions exerted by miRNAs.

### Nature and source of the biological material under investigation

The most obvious limitation here pertains to the use of mice and the (non-)conservation of miRNA and BACE1 mRNA sequences and function between species, as discussed previously [8]. In addition, although very useful for the study of specific aspects of the disease, the animal models of AD that are currently available, in which the disease is caused by altering genes involved in Aß metabolism (eg, mutation of the presenilin 1 gene combined with a chimeric mouse/human APP), only imperfectly mimic and oversimplify a multifactorial disease as complex as AD. In addition, whether the observed changes in miRNA levels in the aging AD brain are the cause or a consequence of the disease (the chicken or the egg dilemma) remains disputable.

Moreover, since the disease is induced "artificially", and does not occur or progress "naturally" in these animals, only the contributory, and not the possible causal, role of miRNAs in the etiology and/or progression of AD can be investigated. For that purpose, the targeted deletion or functional alteration of miRNA function, followed by monitoring of cognitive functions, would be more appropriate.

Animal models may also be more suitable and provide more insights into the pathogenesis of AD progression as compared to humans, where brain tissues may only be obtained, and the data collected, at the time of death, although harvesting brains from subjects of different ages may partially circumvent this issue. In contrast to studies performed in mice, results obtained from post-mortem human brain tissues may be markedly influenced by the time interval between the patients' death and brain tissue harvesting, due to the relatively short half-lives of some miRNAs (~1 to 3.5 h), as recently reported by Sethi and Lukiw [9].

### Use and relevance of cellular models

It is important to underline the utility of cellular models in complementing molecular, biochemical or animal studies. As such, cultured neuronal N2a cells, which have been used to obtain most of the experimental evidences pertaining to the miRNA repression of BACE1 expression [8], are highly relevant and represent the most practical cellular system to study the molecular mechanisms underlying AD.

Whereas specific cell lines, such as 293 or 293T cells, are immortalized upon transformation with adeno-viruses and/or simian virus 40 infection, the cell clone Neuro-2a (N2a) was established from a spontaneous neuroblastoma isolated from the brain of a strain A albino mouse (please refer to http://www.atcc.org). Cytogenetic analysis of these cells revealed an unstable karyotype within a stemline range of 94 to 98 chromo-somes (the cells contain 6 to 10 large chromosomes with median or submedian centromeres and 2 to 4 minute chromosomes). Although N2a cells are of neuronal origin, the endogenous biochemical processes in such cells, that may have undergone tens to hundreds of passages at the time of harvesting, are expected to differ quite markedly from that of primary neuronal cells, thereby limiting the scope of the conclusions that can be reached from their use.

### Use of primary versus cultured cells

The main issue here pertains to two fundamental differences distinguishing primary cells or tissues from immortalized, cultured cells: the genome integrity of the latter and their propensity for cell division. The implication of these differences in the interpretation of miRNA data, which was highlighted not too long ago [10], is major: Previously known as repressors of mRNA translation, miRNAs have also been shown to enhance mRNA translation upon cell cycle arrest [10]. Whether miRNAs function similarly in cell cycle-arrested and primary, non-dividing cells remains to be established. However, the experimental evidences are sufficiently strong to raise a imperative issue as to whether primary, non-dividing cells, such as cholinergic neurons, support miRNA repression and/or enhancement of mRNA translation. This observation also imply that all the miRNA data obtained from cultured cells should be intrepreted with great caution before transposing them to in vivo situations, as miRNAs found to repress specific mRNAs in immortalized cultured cells may exert the exact opposite effects in primary cells or tissues in vivo. Hence to need to obtain data or additional evidences from primary cells that either support or challenge our in vitro miRNA data.

### Other functions exerted by microRNAs

A recent study by Eiring et al. [11] revealed another mean by which miRNAs may enhance gene expression. Reported to regulate BACE1 mRNA translation in the context of AD [8], miR-328 has been shown to have a second function, acting as an RNA decoy by binding to heterogeneous nuclear ribonucleoprotein E2 and lifting its translational repression of an mRNA involved in myeloid cell differentiation [11,12]. Therefore, any decrease or increase in miRNA levels may not yield the expected relief or accentuated repression of gene expression, respectively. This phenomenon may also explain, at least in some cases, the lack of any phenotypic changes associated to specific or global miRNA variations, both in terms of levels and mode of action, the cumulative effects of which may cancel each other out.

Another level of complexity may be conferred by the ability of miRNAs to regulate multiple mRNA targets, to exert indirect effects and to be involved in more complex networks of regulatory mediators of importance in the pathogenesis of age-related diseases like AD.

## Conclusion and perspectives

Recently, the major research advances pertaining to the possible role and function of miRNAs in neurodegenerative diseases, such as AD, has provided novel perspectives to the pathogenesis of increasingly prevalent, age-related diseases in human. However, further investigations are required in order to improve our understanding of the changes that may occur in miRNA biogenesis, metabolism and/or function during aging, which may be an important contributor to the etiology and progression of age-related diseases. In this regard, several issues related to the impact of aging and/or cell division on the integrity and functionality of the miRNA pathway remain to be explored and might add an additional layer of complexity to miRNA studies involving mRNA translational regulation: Is the biogenesis and/or function of miRNAs modified as the cells are dividing, or altered by age-related processes? Are miRNA genes shut off or turned on, either specifically or globally, during aging? Do cells acquire or lose functional miRNAs as they age? As they divide? In that context, prudence and cautiousness should guide us when interpreting and extrapolating experimental findings related to miRNAs to a human disease in particular. In AD, for instance, primary human brain tissues obtained upon death of AD and non-AD patients remain the most relevant source of biological materials in order to get further insights into the pathogenesis of AD. However, the utility, versatility and complementarity of animal and cell culture models, albeit imperfect and coming with their pros and cons, cannot be ignored, as the insights they provide simply need to be considered into their proper context with their own limitations and promises.
